# Evaluation of a new foetal shielding device for pregnant brain tumour patients

**DOI:** 10.1186/s13014-021-01836-z

**Published:** 2021-06-13

**Authors:** Seonghee Kang, Kyeong-Hyeon Kim, Sang-Won Kang, Dong-Seok Shin, Seungwan Lee, Jin-Beom Chung

**Affiliations:** 1 Department of Radiation Oncology, Seoul National University Bundang Hospital, 82 Gumi-ro 173 Beon-gil, Bundang-gu, Seongnam-si, 13620 Gyeonggi-do Korea; 2 Department of Biomedical Engineering, Research Institute of Biomedical Engineering, College of Medicine, The Catholic University of Korea, Seoul, Korea; 3 Department of Radiological Science, Konyang University, Daejeon, Korea; 4 Present Address: Department of Radiation Oncology, Seoul National University Hospital, Seoul, Republic of Korea

**Keywords:** Foetal dose, Foetal shielding device, VMAT, Pregnant patient

## Abstract

**Background:**

The present study aimed to propose a new foetal shielding device for pregnant cancer patients to reduce the foetal dose associated with treatment techniques using multiple gantry angles, such as intensity-modulated radiation therapy (IMRT) or volumetric modulated arc therapy (VMAT).

**Methods:**

Three shielding structures were designed to minimise the scattered and leaked radiation from various gantry angles and radiation scattering within the patient. The base-plate part that can be placed on the treatment couch was designed to reduce the scattered and leaked radiation generated at gantry angles located near 180°. A body shielding part that can cover the lower chest and abdomen was designed, and a neck-shielding structure was added to reduce the internal and external radiation scattering from the treatment area. Evaluation plans were generated to assess the foetal dose reduction by the foetal shielding device in terms of the shielding material thickness, distance from the field edge, and shielding component using the flattened 6 MV photon beam (6MV) and flattening filter-free 6 MV photon beam (6MV-FFF). In addition, the effectiveness of the foetal shielding device was evaluated in a pregnant brain tumour patient.

**Results:**

The shielding material consisting of three parts was placed on frames composed of four arch shapes with a vertical curved structure, connection bar at the top position, and base plate. Each shielding part resulted in reductions in the radiation dose according to the treatment technique, as the thickness of the shielding material increased and the foetal dose decreased. In addition, a foetal dose reduction of approximately 50% was confirmed at 50 cm from the field edge by using the designed shielding device in most delivery techniques. In patients, the newly designed shielding structures can effectively eliminate up to about 49% of the foetal dose generated from various gantry angles used in VMAT or IMRT.

**Conclusions:**

We designed a foetal shielding device consisting of three parts to effectively reduce the dose delivered to the foetus, and evaluated the device with various treatment techniques for a pregnant patient with brain tumour. The foetal shielding device shielded the scattered/leaked radiation from the treatment machine, and also effectively reduced internal scattering from the treatment area in the patient.

## Background

Although radiation therapy is rarely performed for pregnant patients, it has been used for pregnant patients with breast, head and neck cancer, Hodgkin’s disease, leukaemia, and brain tumour [1–3]. For such patients, it is important to consider target control and saving of the surrounding organs at risk (OARs) as well as foetal dose reduction. When radiation therapy for a pregnant patient is determined, a suitable treatment strategy and shielding structures to mitigate the potential risk to the foetus by reducing the peripheral dose should be considered [1, 4]. As recommended by the American Association of Physicists in Medicine Task Group 36 (AAPM TG-36), the dose delivered to the foetus should be maintained below 5 cGy to minimise the adverse biological effects of the radiation, which depend on various factors such as gestational age, equivalent dose, and radiation type [1]. In external beam radiotherapy, the foetal dose can be attributed to radiation leakage from the head of the linear accelerator (LINAC) and scatter from the collimator, blocks, and other objects. To minimise the foetal dose, the distance from the radiation field edge should be kept as far as possible, and an optimum treatment technique with an appropriate field size and beam angles should be used [5–8].

When using techniques with relatively large modulation, such as volumetric modulated arc therapy (VMAT) or intensity-modulated radiation therapy (IMRT), to improve target coverage and normal tissue sparing, the dose delivered to the foetus may be increased by the scattered radiation from various angles. Various treatment strategies such as 3D-conformal radiation therapy (3D-CRT), IMRT, and tomotherapy have been compared with respect to their ability to reduce the foetal dose [9–12]. David et al. [13] reported that the foetal dose from radiotherapy of glioblastoma during pregnancy can be reduced with IMRT by using a mobile shielding device. Owrangi et al. [8] proposed a custom foetal shield to allow multiple beam angles, and the peripheral dose (PD) measurement was evaluated with and without the shielding structures. However, the previously proposed shielding designs were not sufficient to adequately shield the doses scattered by patients and some delivery techniques, such as VMAT or tomotherapy. In addition, addition or removal of shielding materials such as lead to provide an effective foetal dose shield was difficult.

In patients with Hodgkin’s disease, where the distance between the treatment field and the foetus is low, three to five half-value layers (HVLs) of lead should be used to sufficiently shield the dose delivered to the foetus [1, 14]. However, in patients with brain or head and neck (H&N) cancer, wherein the distance of the foetus from the field edge would be more than 30 cm, VMAT or IMRT might be considered as a treatment option to improve the therapeutic gain. We designed a new shielding structure for brain and H&N cancer patients to reduce the dose delivered to the foetus for various treatment techniques using multiple gantry angles. Shielding structures consisting of three parts were designed to effectively reduce the dose delivered to the foetus, and the difference in the foetal dose with or without shielding was evaluated using a modified Rando-phantom to verify the effectiveness of the shielding structures.

## Methods

### Shielding structure design

**Fig. 1:**
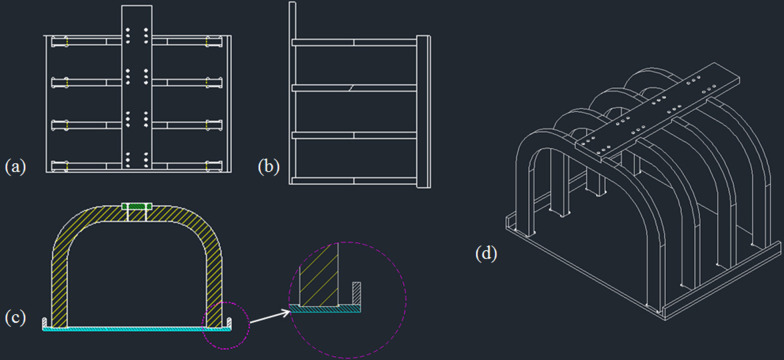
Schematic diagram of the frame for the shielding material: **a** top view, **b** side view, **c** frontal view, and **d** the assembled foetal shielding device to support the shielding material

The principal sources for the dose delivered to the foetus are (1) treatment head leakage from the LINAC, (2) scattered radiation from the collimators and beam modifiers, and (3) radiation scattered within the patient from the treatment beam. In this study, shielding structures were designed to reduce the doses from these three sources during treatment. In particular, because VMAT can generate scattered or leakage radiation from a continuously rotating gantry head, effective shielding of these sources is essential. Therefore, three shielding structures were designed to minimise the scattered and leaked radiation from various gantry angles and the scattered radiation within the patient. Figure 1 shows schematic diagram of the frames to fabricate the shielding structures. First, the part A for neck shielding was added to reduce the internal and external scattered radiation from the treatment area. The internal scattered radiation generated at the target and directed out of the neck can be shielded with the part A. The part B for body shielding that can cover the lower chest and abdomen was designed. In addition, the part C of base-plate that can be placed on the treatment couch was designed to reduce the scattered and leaked radiation generated at gantry angles located near 180°. The frame was used to prevent the deformation and deflection of the shielding structure fabricated with lead.

### Evaluation of the foetal shielding device

**Fig. 2:**
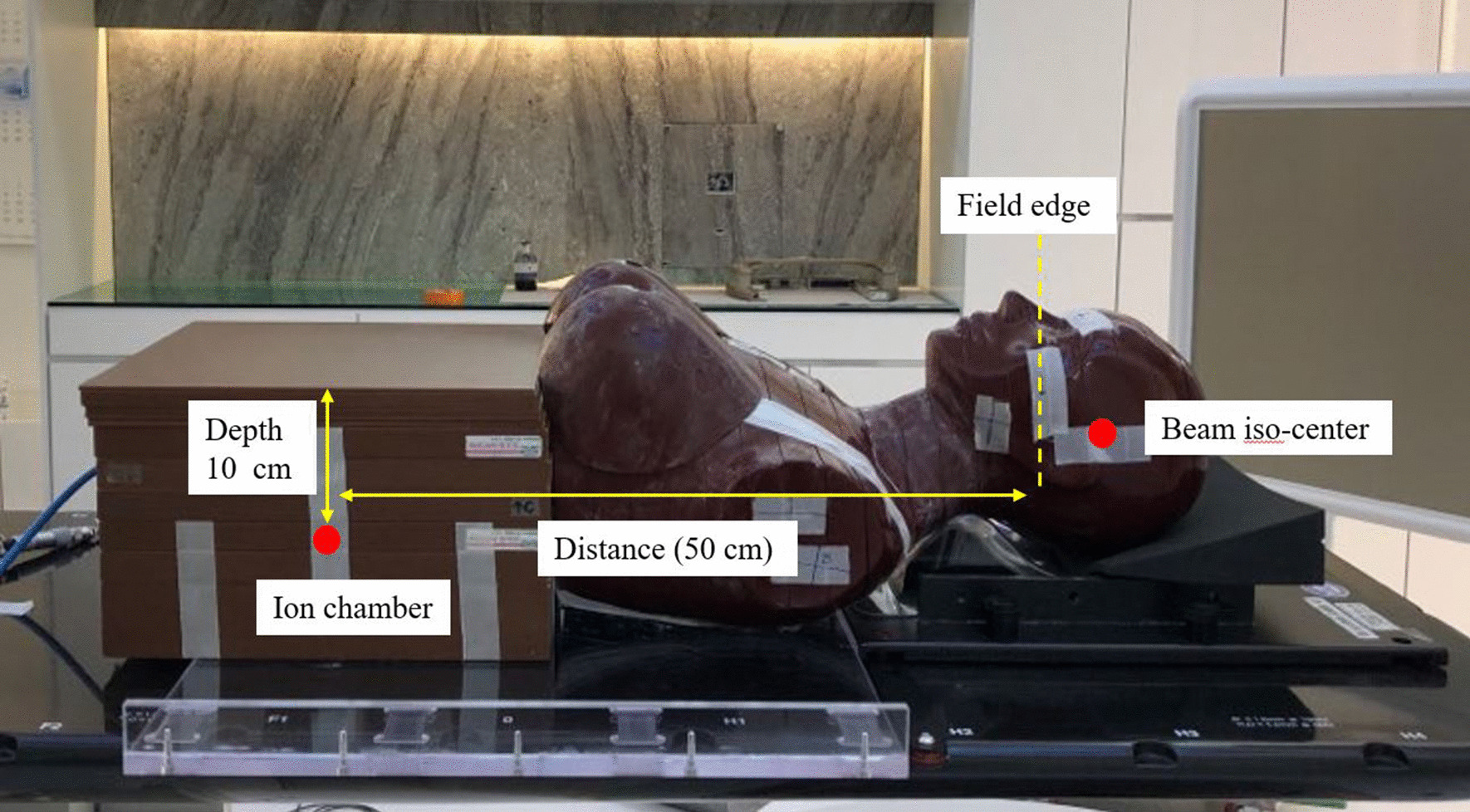
The modified Rando-phantom set-up for PD measurement. The modified phantom consisted of a Rando-phantom attached to a 30 × 30 × 20 cm^3^ solid water phantom

Evaluation plans (VMAT plan with two full arcs and IMRT plan arranged with seven gantry angles) were generated to evaluate the PD reduction by the shielding structures in a rotational delivery technique using a flattened 6-MV photon beam (6 MV) and a flattening filter-free 6-MV photon beam (6 MV FFF), as shown in Table 1. The prescription dose was 60 Gy, with a daily dose of 2 Gy. All treatment plans were created using the photon optimizer (PO; Varian Medical Systems, Palo Alto, CA) and the anisotropic analytic algorithm (AAA; Varian Medical Systems, Palo Alto, CA). The grid size used for dose calculation was 2.5 mm. Dose deliveries for all plans were performed with TrueBEAMTM using a 2.5-mm high-definition multi-leaf collimator (HD MLC, Varian Medical Systems, Palo Alto, CA). The virtual brain tumour in the modified Rando-phantom was 120 cc (equivalent sphere diameter: 6 cm), and at least 95% of the planning target volume (PTV) was covered with 100% of the prescription dose. Figure 2 shows the modified Rando-phantom set-up to measure the PD generated during treatment. Since the treatment couch lift capacity should be considered, the appropriate thickness of the shielding material was determined by assessing 6–12-mm lead with a 2-mm thickness interval. In addition, the effectiveness of each component was evaluated by measuring various combinations for each component with VMAT and IMRT plans, and measurements were performed to obtain the doses at a depth of 10 cm at 30 cm, 40 cm, and 50 cm distance from field edge to evaluate the dose reduction by the shielding device according to distance. Three repeated measurements were performed using a 0.6-cm^3^ Farmer-type ionisation chamber (Waterproof PTW Farmer Chamber type 30013, Freiburg, Germany), and the measured foetal doses were estimated in terms of average value. The chamber and electrometer were carefully operated to minimise leakage during the measurement.

**Table 1:** Treatment plan for foetal dose evaluation

Energy	Gantry angle (°)	MU	Delivery time (s)
VMAT			
6 MV	2 full arcs	270/265	144
6 MV FFF	2 full arcs	272/303	141
IMRT			
6 MV	50/100/140/180/220/260/310	79/89/55/63/52/86/82	185
6 MV FFF	50/100/140/180/220/260/310	123/118/79/91/85/111/121	183

*VMAT* volumetric modulated arc therapy, *IMRT* intensity-modulated radiation therapy, *FFF* flattening filter-free

### Patient case

The patient was 23 weeks pregnant when she was diagnosed with left ventricle glioblastoma (T2 and grade 4) and received postoperative radiotherapy in our department. Computed tomography (CT) simulation was performed to generate a treatment plan with Brilliance CT Big BoreTM (Philips, Cleveland, OH, USA) with a 2-mm slice-thickness. The CT scan range for treatment planning was 36 cm, including head to neck. The patient was immobilised using an Aquaplast facemask (WFR Aquaplast, Wyckoff, NJ). T1 contrast-enhanced and T2 fluid‐attenuated inversion recovery (FLAIR) magnetic resonance imaging (MRI) was used to define the target volume, and CT images were imported into the Eclipse treatment planning system (Ver. 13.7, Varian Medical Systems, Palo Alto, CA) for treatment planning. Dose calculation was performed by using the analytic anisotropic algorithm (AAA) with heterogeneity correction and a dose calculation gird size of 2.5 mm. A dose of 60 Gy in 30 fractions was prescribed to the PTV. The primary goal of treatment planning was to cover at least 100% of the PTV with 95% of the prescribed dose. Table 2 shows the maximum dose constraint for clinical OARs. A VMAT plan with two partial arcs and an IMRT plan arranged with 7 gantry angles were used to measure the PD reduction by the shielding structures. To maximally separate the treatment site from the foetus position, the treatment couch was not rotated, and the vertex beam, which could deliver the primary beam directly to the foetus, was not used. In addition, for IMRT, the collimator was rotated 90° to place the distal × jaws in the superior-inferior direction for the patient. The treatment plans were also generated using the PO and AAA, and TrueBEAMTM using HD MLC was used for beam delivery. Since the distance between the field edge and the umbilicus of the patient is approximately 40 cm, three measurements were performed using a 0.6-cm^3^ farmer-type ionisation chamber at a 10-cm depth with a 40-cm distance from the field edge corresponding to the distance of the umbilicus. In addition, three points have been specified as distances from the field edge (30 cm for fundus, 40 cm for umbilicus, 50 cm for pubis) according to the TG 36 recommendation, and dose measurements have been performed by using the farmer-type ionisation chamber placed under the 5 mm bolus at the surface of the phantom. Breasts dose were measured by using the ionisation chamber placed 7 cm laterally from the central axis and 20 cm inferiorly from the field edge. The 5 mm boluses were place under and upper the ionisation chamber.

**Table 2:** Clinical OAR dose constraints

Critical structure	Dose constraint
Chiasm	Dmax < 55 Gy to whole structure (< 3%)
Left optic nerve	Dmax < 55 Gy to whole structure (< 3%)
Right optic nerve	Dmax < 55 Gy to whole structure (< 3%)
Brainstem	Dmax < 54 Gy to whole structure (< 5%)
Left eye	Dmax < 15 Gy to whole structure
Right eye	Dmax < 15 Gy to whole structure
Left hippocampus	Dmax < 12 Gy to 20% of structure
Right hippocampus	Dmax < 12 Gy to 20% of structure

## Results

### Foetal shielding device

**Fig. 3:**
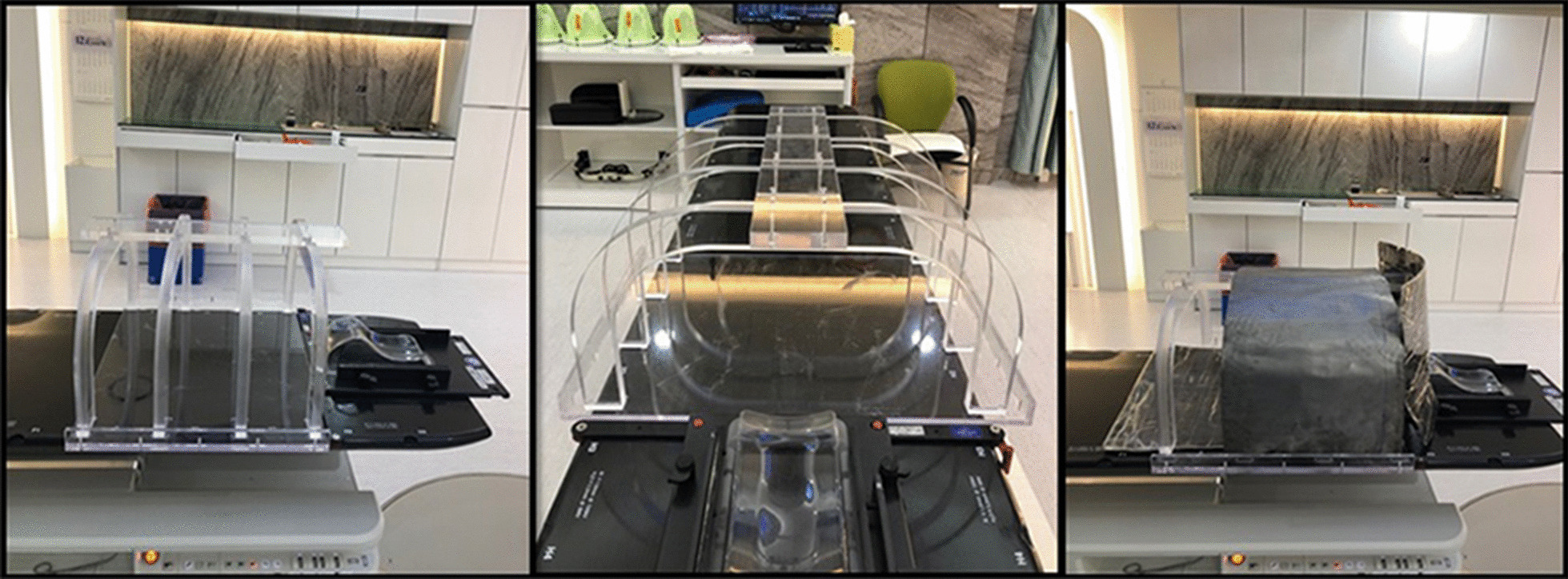
Photo of the foetal shielding devices with shielding materials (lead)

As shown in Fig. 3, frames that can support the shielding materials were fabricated. The three parts of the shielding material were placed on frames composed of four arch shapes with a vertical curved structure, a connection bar at the top position, and a base plate. The arch-shaped frame was made of acrylic with a 5-cm thickness to withstand the weight of the shielding materials, and the height and width of the inner cavity were 30 cm and 46 cm, respectively. The connection bar was designed to change the position of the four arch shaped frames to suit the patient’s body shape, and it could support the neck shielding material using the protruding part. The height and width of the inner cavity could be adjusted by using frames of various sizes according to the patient’s body size. The base plate was manufactured to be combined with the four arch-shaped frames, and a 2-cm barrier was attached to prevent shielding materials from escaping outside the base plate.

### Effectiveness of the foetal shielding device

Table 3 shows the relative percentage of foetal dose reduction with and without the foetal shielding device to evaluate the shielding effect of each component. Foetal dose measurement was performed 30 cm away from the field edge using 10-mm lead. In the VMAT technique, all combinations except part C and parts A + B + C were confirmed to show a greater dose reduction at 6 MV FFF than at 6 MV. Among parts A, B, and C, the foetal dose reduction was greater at part C at 6 MV (23.58%) and 6 MV FFF (23.16%) in the VMAT. When using only two parts, the reduction was greater at 6 MV FFF than at 6 MV; on the other hand, when using all parts, the foetal dose decreased more at 6 MV. In IMRT, unlike the VMAT, greater reductions were observed at 6 MV, except for part A and parts A + C; 6 MV and 6 MV FFF showed greater reductions of 26.41% and 22.22% with part B in comparison with parts A and C. The maximal foetal dose reduction was confirmed at 6 MV with parts A + B in comparison with the other two pairs of parts, but greater foetal dose reduction was also achieved by using parts A + B + C at 6 MV than 6 MV FFF.

**Table 3:** Relative foetal dose reduction rate with and without each part of 10 mm shielding device. The point of measurement is from 30 cm at the field edge and 10 cm depth from the surface

	Part A (%)	Part B (%)	Part C (%)	Parts A + B (%)	Parts A + C (%)	Parts B + C (%)	Parts A + B + C (%)
VMAT							
6 MV	17.92	20.75	23.58	26.42	29.25	31.13	36.79
6 MV FFF	21.05	22.11	23.16	28.42	30.53	31.58	35.79
IMRT							
6 MV	21.50	26.41	15.37	31.31	22.73	22.73	34.99
6 MV FFF	22.22	22.22	13.58	25.93	25.93	20.99	33.77

*VMAT* volumetric modulated arc therapy, *IMRT* intensity-modulated radiation therapy, *FFF* flattening filter-free, *Part A* neck shielding, *Part B* body shielding, *Part C* back shielding

In order to evaluate the appropriate thickness of shielding materials, the foetal dose reduction with all parts of the foetal shielding device at 2-mm thickness intervals from 6 to 12 mm are shown in Table 4. The reduction was greater at 6 MV than at 6 MV FFF for both VMAT and IMRT for all thicknesses. As expected, the foetal dose decreased with an increase in the shielding material thickness. For 12-mm lead, a maximal reduction from 2.83 to 1.75 cGy was observed with 6 MV VMAT, whereas the least reduction from 2.16 to 1.41 cGy was observed with 6 MV FFF IMRT. Interestingly, the difference in the extent of reduction between two successive thicknesses was the smallest (0.5%) between 10 and 12-mm lead with the 6 MV FFF IMRT, and the largest (5.9%) between 6 and 8-mm lead with the 6MV IMRT. The difference between 6 and 6 MV FFF in VMAT was 0.18 cGy, showing the largest difference for 6-mm lead, whereas in IMRT, the smallest difference of 0.01 cGy was found with 10-mm lead.

**Table 4:** Foetal dose reduction at a point 30 cm away from the field edge for different thicknesses of shielding material with parts A + B + C in comparison with no shielding device

	W/O FSD (cGy)	6-mm lead (cGy)	8-mm lead (cGy)	10-mm lead (cGy)	12-mm lead (cGy)
VMAT					
6 MV	2.83	2.02	1.89	1.77	1.75
6 MV FFF	2.53	1.84	1.73	1.61	1.59
IMRT					
6 MV	2.19	1.57	1.44	1.41	1.36
6 MV FFF	2.16	1.62	1.54	1.42	1.40

*VMAT* volumetric modulated arc therapy, *IMRT* intensity-modulated radiation therapy, *FFF* flattening filter-free, *FSD* foetal shielding device, *W/O* without

Table 5 shows the foetal doses measured at various distances from the field edge using 10-mm lead with all parts. The dose could be reduced by approximately 50% at 50 cm from the field edge by using the foetal shielding device in most delivery techniques. This confirmed that as the distance increased from 30 to 50 cm, the reduction in the foetal dose with the shielding device increased. In comparison with other delivery techniques, when 6 MV VMAT was used, foetal doses measured at 30 cm and 40 cm from the edge of the field showed the greatest reduction, and the least reduction was observed in 6 MV FFF IMRT at the 30-cm distance. On the other hand, the greatest dose reduction at 50 cm from the field edge was achieved with 6 MV FFF VMAT, and the smallest dose reduction at the same distance was achieved with 6 MV IMRT.

**Table 5:** Foetal doses measured at points at various distances from the field edge with and without the foetal shielding device using parts A + B + C with 10-mm lead

	W/O FSD at 30 cm (cGy)	With FSD at 30 cm (cGy)	W/O FSD at 40 cm (cGy)	With FSD at 40 cm (cGy)	W/O FSD 50 cm (cGy)	With FSD at 50 cm (cGy)
VMAT						
6 MV	2.83	1.77	1.39	0.87	1.17	0.59
6 MV FFF	2.53	1.61	1.12	0.71	0.77	0.37
IMRT						
6 MV	2.19	1.41	1.20	0.77	1.09	0.59
6 MV FFF	2.16	1.42	0.99	0.64	0.80	0.48

*VMAT* volumetric modulated arc therapy, *IMRT* intensity-modulated radiation therapy, *FFF* flattening filter-free, *FSD* foetal shielding device, *W/O* without

### Patient case

**Fig. 4:**
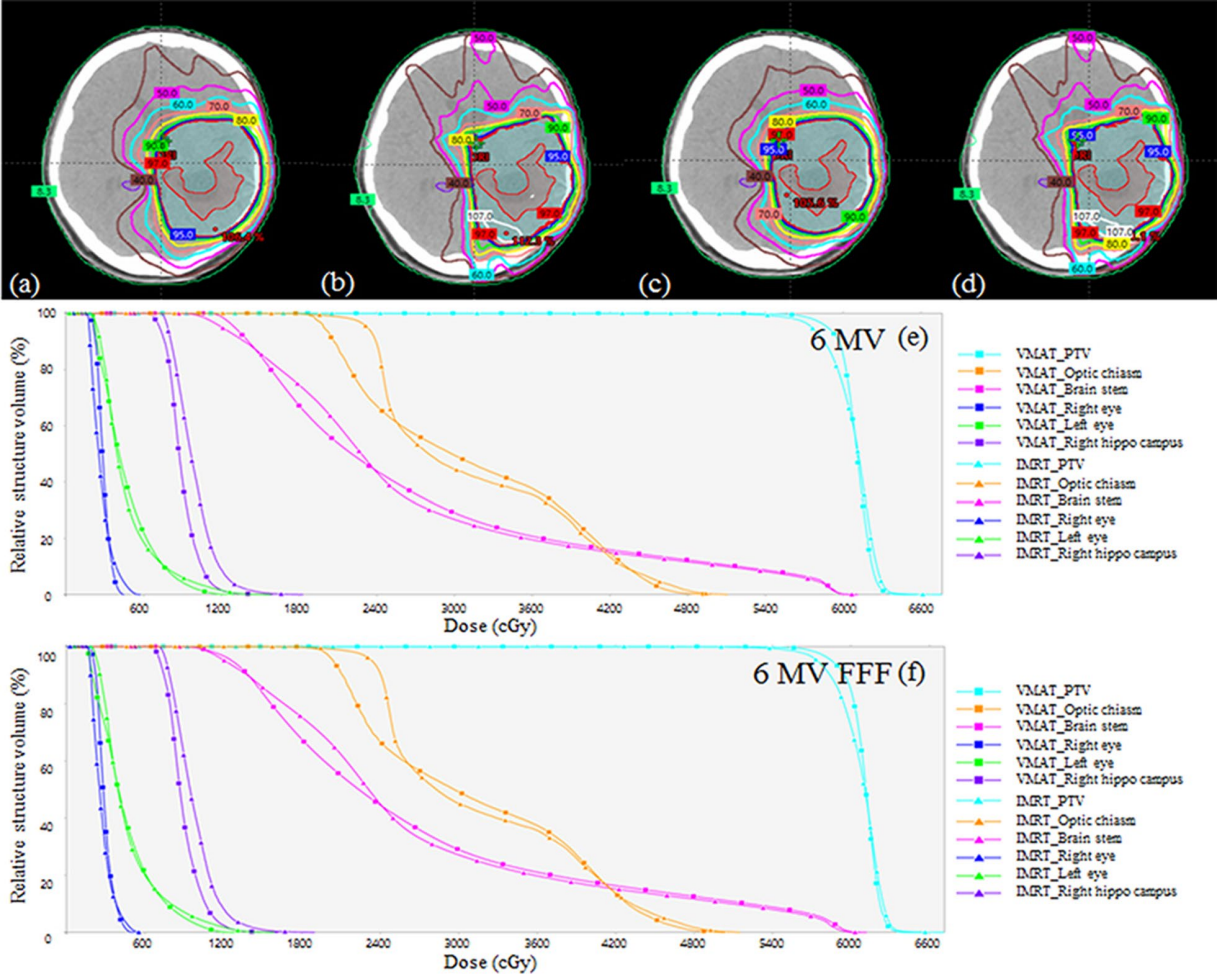
Dose distributions and DVHs of various treatment techniques for the patient: **a** 6 MV VMAT, **b** 6 MV IMRT, **c** 6 MV FFF VMAT, **d** 6 MV FFF IMRT, **e** DVH for 6 MV VMAT and IMRT, and **f** DVH for 6 MV FFF VMAT and IMRT

Figure 4 shows the dose distributions and dose–volume histograms (DVHs) of various treatment plans that met the clinical objective for target volume and critical structures for brain tumour patients. In comparison with the IMRT plans, the VMAT plans showed better dose distribution and PTV coverage. Table 6 indicates the measured foetal dose at a point approximately 40 cm away from the field edge at the depth of 10 cm with and without the foetal shielding device using 10-mm lead. The measurement was carefully carried out by monitoring the leakage dose during each measurement, and dose values were corrected for pressure and temperature. Foetal dose in 6 MV FFF IMRT reduced the most to 48.78% with a foetal shielding device, whereas the lowest decrease was 44.44% in the 6 MV FFF VMAT. The highest dose was measured at 6 MV VMAT, and the lowest dose was measured in 6 MV FFF VMAT. Table 7 shows the fundus, umbilicus and symphysis pubis point dose measurement by using the ionisation chamber. All measurement points reduced under 5 cGy with a foetal shielding device, and the dose reductions of the VMAT plans were higher than those of the IMRT plans. The measured breasts dose with and without the foetal shielding device are presented in Table 8. Dose reductions of more than 42% in the IMRT plans and those of more than 45% in the VMAT plans were observed.

**Table 6:** The measured foetal doses with and without the foetal shielding device using all parts in a pregnant patient plan at a distance of 40 cm and 10 cm depth from surface

	FD without FSD (cGy)	FD with FSD (cGy)	Difference (%)
VMAT			
6 MV	3.68	2.00	45.65
6 MV FFF	2.88	1.60	44.44
IMRT			
6 MV	3.26	1.84	45.24
6 MV FFF	3.28	1.68	48.78

*VMAT* volumetric modulated arc therapy, *IMRT* intensity-modulated radiation therapy, *FFF* flattening filter-free, *FSD* foetal shielding device, *FD* foetal dose

**Table 7:** The measured foetal doses with and without the foetal shielding device using all parts in a pregnant patient plan at the three point represented by fundus, umbilicus and symphysis pubis

	Fundus		Umblicus		Symphysis pubis	
	W/O FSD at 30 cm (cGy)	With FSD at 30 cm (cGy)	W/O FSD at 40 cm (cGy)	With FSD at 40 cm (cGy)	W/O FSD 50 cm (cGy)	With FSD at 50 cm (cGy)
VMAT						
6 MV	8.882	4.764	3.876	1.938	2.019	0.969
6 MV FFF	7.267	4.037	3.149	1.534	1.534	0.727
IMRT						
6 MV	7.267	4.118	4.279	2.019	3.795	2.019
6 MV FFF	6.217	3.876	3.472	1.696	2.826	1.534

*VMAT* volumetric modulated arc therapy, *IMRT* intensity-modulated radiation therapy, *FFF* flattening filter-free, *FSD* foetal shielding device, *FD* foetal dose

**Table 8:** The measured breasts dose with and without the foetal shielding device using all parts in a pregnant patient plan

	Right breast		Left breast	
	W/O FSD	With FSD	W/O FSD	With FSD
VMAT				
6 MV	18.651	10.254	19.378	10.335
6 MV FFF	16.148	8.801	16.794	8.478
IMRT				
6 MV	16.310	9.204	15.099	8.478
6 MV FFF	15.179	8.720	13.968	8.074

*VMAT* volumetric modulated arc therapy, *IMRT* intensity-modulated radiation therapy, *FFF* flattening filter-free, *FSD* foetal shielding device, *FD* foetal dose

## Discussion

Radiation therapy for pregnant patients should aim to maximise effective and accurate therapeutic effects while minimising the dose delivered to the foetus. Because even small doses delivered to the foetus can be associated with a potentially significant risk, additional care must be taken to confirm to the as low as reasonably achievable (ALARA) principle. According to the AAPM TG Report 36, the risks of radiation to a foetus are classified into seven categories, and adverse biological effects are influenced by various factors such as absorbed dose, type of radiation, and the gestational age at exposure [1]. Once a treatment strategy has been determined, it is necessary to determine whether additional shielding is required and, if necessary, design shielding structures accordingly.

VMAT usually demonstrates superiority to other delivery techniques in terms of the DVH and dose distribution. However, it is important to thoroughly review the effects of high-modulation techniques such as VMAT or IMRT on foetal dose, since such therapies can increase scattered and leakage doses. The previous shielding structures were mostly designed to shield the upper part of the patient; few shielding structures were proposed to shield the scattered and leakage doses that occur when gantry is located at around 180° and for the internal scattered radiation [1, 3, 4, 8]. Thus, the previously designed structures would not be sufficient for VMAT or IMRT using gantry angles of approximately 180°. In addition, shielding against internal scattering consisting of low-energy Compton-scattered photons should be considered to reduce the peripheral dose. We designed shielding structures composed of three parts to effectively shield the radiation generated from a continuous gantry angle. First, the neck shielding structure (part A) is used to minimise the internal scattered radiation caused by the Compton scattered photon, which is approximately 500 keV [15]. The shielding structure for internal scattering is sufficiently wide to shield the scattered radiation by considering the divergence of the scattered and leakage radiation. Because the shape of the body shielding part made by lead can be changed owing to the characteristics of lead, which is soft and malleable, the four acrylic frames were manufactured to minimise the change in shape. The body frame, which is 5-cm-thick, can support heavy lead and provides enough inner cavities for the patient. Lastly, the base plate was made using acrylic panels, and the patient was set up on the base plate placed on the treatment couch. Using these shielding devices, the gantry head scatter and leakage radiation occurring around all directions can be effectively reduced by fully shielding with lead from all sides of the patient.

By using all parts in IMRT and VMAT techniques at a point 30 cm from the edge, the foetal dose was reduced by up to about 37%, and when using only two parts, namely, A + B, A + C, and B + C, the reductions were up to 31.31%, 30.53%, and 31.58%, respectively. Thus, omission of one part can result in up to about 12% greater foetal doses. Since the weight allowance of the Exact IGRT couch (Varian Medical Systems, Palo Alto, CA) was approximately 227 kg, the overall weight of the foetal-shielding should be less than 100 kg. The total weight of the baseplate, arch shape frame, connection bar and lead may be approximately 92 kg, which not exceed 100 kg. In accordance with the TG-36 report, a lead thickness of 5 to 7 cm is sufficient to reduce the PD dose regardless of the energy, but other studies have reported that shielding structures with a smaller thickness were sufficient in a low-energy photon beam [1, 16, 17]. In our study, we used only 6 MV and 6 MV FFF beams, not more than 10 MV, to prevent photo-neutron generation. Therefore, it is necessary to determine the appropriate thickness of the shielding device considering the beam energy, treatment technique, and the weight of the shielding materials. As the thickness of the shielding devices increased, the foetal dose decreased more, but 10-mm lead, which can achieve a dose of less than 5 cGy, was used in our study. By using 10-mm lead, it is possible to reduce couch sagging as well as improve the convenience of the patient setup. Owrangi et al. [8] reported that PD decreased as the distance between the treatment site and the measurement point increased. Consistent with their findings, as the distance increased, the foetal dose reduction caused by the foetal shielding device also increased. Particularly, the fundus doses without shielding device were more than 5 cGy in both VMAT and IMRT plans. These are higher than the recommendation of AAPM TG-36, suggesting that the fundus dose should be controlled. However, dose under the 5 cGy were measured at the all point of measurement by using the proposed fetal shielding device. As the pregnancy week increases, the fundus moves upward [1]. Therefore, it would be necessary to increase the shielding thickness depending on the pregnancy week. In the patient case, 6 MV VMAT was performed using the smallest monitor units (MU) and beam delivery time (81 s), but it showed the largest foetal dose. On the other hand, the smallest foetal dose was delivered in 6 MV FFF VMAT, and the beam delivery time was 83 s, similar to the 6 MV VMAT. This is due to the reduction in head leakage caused by the flattening filter, and approximately 20% reduction in foetal dose in VMAT using 6 MV FFF compared to 6 MV was observed [3]. The average beam delivery time in IMRTs was approximately 230 s, which was increased by approximately 35% compared to the 6 MV FFF VMAT, and a higher foetal dose was measured. The foetal dose measured in the patient was less than 5 cGy using the proposed foetal shielding device for all treatment techniques. In addition, the breast dose caused by scattered radiation can be reduced by the shielding device. However, it is necessary to assess more patient cases, and further studies on the applicability of these devices to patients with head and neck cancers as well as various brain tumours are required.

## Conclusions

We created a three-part foetal shielding device to effectively reduce the dose delivered to the foetus and evaluated a variety of treatment techniques for a pregnant patient with brain tumour. The newly developed shielding devices were able to effectively eliminate scattered/leakage radiation generated at various gantry angles used in VMAT or IMRT. This shielding device can be easily adapted to the patient to minimise the peripheral dose in many other situations such as CT simulation, imaging dose for patient setup, and radiation therapy for young patients. Using the 6 MV FFF VMAT technique, better dose distribution and shorter delivery time were confirmed, and when applied with the shielding structures, the lowest foetal dose could be delivered. This approach may help reduce adverse effects by minimising the doses delivered to the foetus while effectively treating pregnant patients with the latest treatment techniques.

## Data Availability

The datasets supporting the study conclusions are included within this manuscript.

## Abbreviations

OARs: Organs at risk
AAPM TG-36: American Association of Physicists in Medicine Task Group 36
LINAC: Linear accelerator
IMRT: Intensity-modulated radiation therapy
VMAT: Volumetric modulated arc therapy
3D-CRT: 3D-conformal radiation therapy
PD: Peripheral dose
HVLs: Half-value layers
H&N: Head and neck
6 MV: 6 MV photon beam
6 MV-FFF: Flattening filter-free 6 MV photon beam
PO: Photon optimizer
AAA: Anisotropic analytic algorithm
HD MLC: High-definition multi-leaf collimator
PTV: Planning target volume
CT: Computed tomography
FLAIR: Fluid‐attenuated inversion recovery
MRI: Magnetic resonance imaging
DVHs: Dose–volume histograms
ALARA: As low as reasonably achievable
MU: Monitor units
